# A CACNA1A variant associated with trigeminal neuralgia alters the gating of Cav2.1 channels

**DOI:** 10.1186/s13041-020-00725-y

**Published:** 2021-01-07

**Authors:** Eder Gambeta, Maria A. Gandini, Ivana A. Souza, Laurent Ferron, Gerald W. Zamponi

**Affiliations:** Department of Physiology and Pharmacology, Alberta Children’s Hospital Research Institute, Hotchkiss Brain Institute, Cumming School of Medicine, University of Calgary, Calgary, Canada

**Keywords:** P/Q channel, Calcium channel, Facial pain, Ion channel, Electrophysiology

## Abstract

A novel missense mutation in the *CACNA1A* gene that encodes the pore forming α_1_ subunit of the Ca_V_2.1 voltage-gated calcium channel was identified in a patient with trigeminal neuralgia. This mutation leads to a substitution of proline 2455 by histidine (P2455H) in the distal C-terminus region of the channel. Due to the well characterized role of this channel in neurotransmitter release, our aim was to characterize the biophysical properties of the P2455H variant in heterologously expressed Ca_V_2.1 channels. Whole-cell patch clamp recordings of wild type and mutant Ca_V_2.1 channels expressed in tsA-201 cells reveal that the mutation mediates a depolarizing shift in the voltage-dependence of activation and inactivation. Moreover, the P2455H mutant strongly reduced calcium-dependent inactivation of the channel that is consistent with an overall gain of function. Hence, the P2455H Ca_V_2.1 missense mutation alters the gating properties of the channel, suggesting that associated changes in Ca_V_2.1-dependent synaptic communication in the trigeminal system may contribute to the development of trigeminal neuralgia.

## Introduction

Trigeminal neuralgia (TN) is one of the most common forms of craniofacial pain. TN is defined as sudden unilateral severe short-lasting pain in one or more branches of the trigeminal nerve [1]. The etiology of TN can be classified into (1) classical TN, which is related to neurovascular compression in the root entry zone at the trigeminal ganglia, (2) secondary TN which is associated with neurological diseases such as multiple sclerosis, and (3) idiopathic TN with unknown etiology [2]. With an incidence of 4–13 cases per 100,000 people, it has been reported that TN can exhibit familial linkage [3], suggesting that some forms of TN may have a genetic component that could be polygenic and multifactorial.

**Fig. 1:**
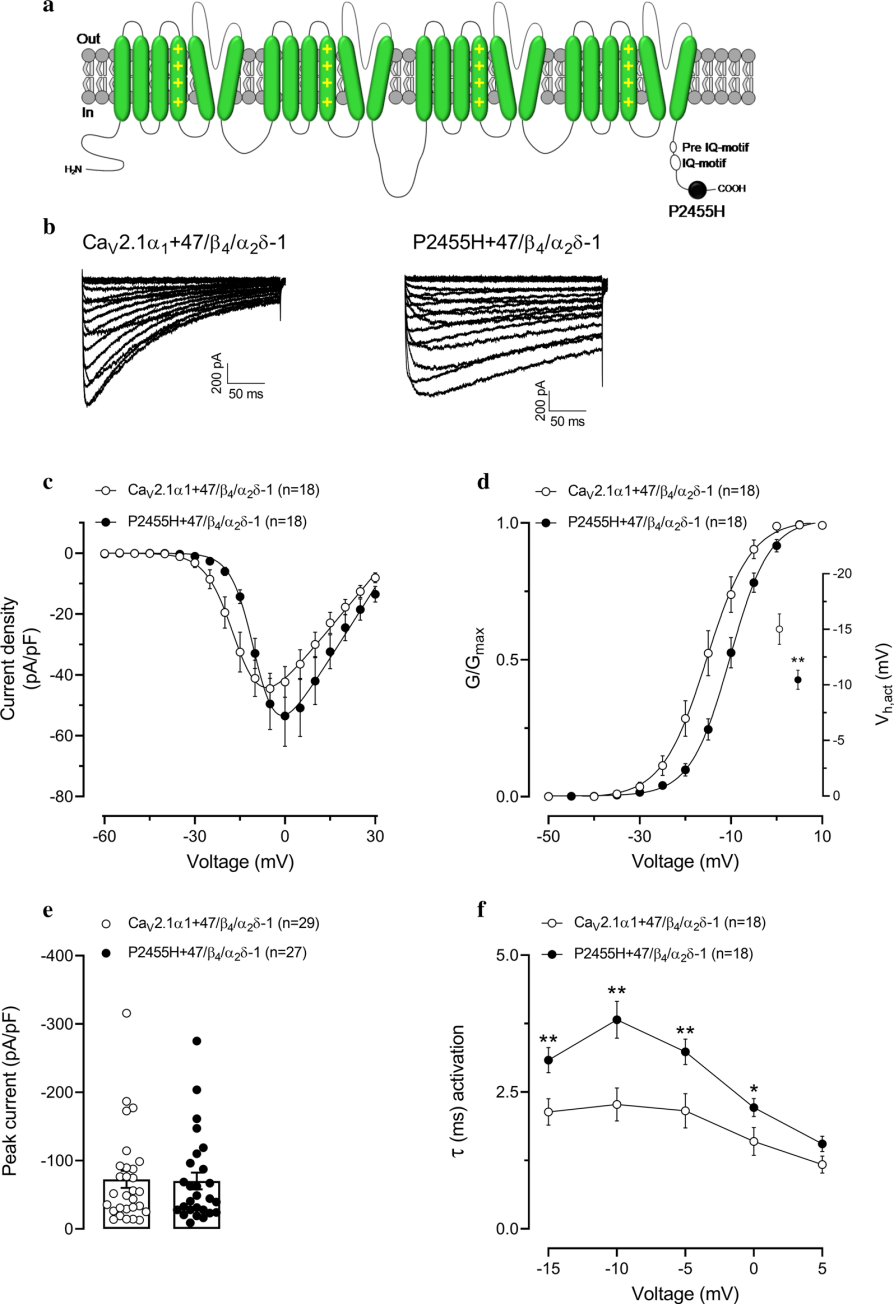
Functional effects of the P2455H mutation on channel activation. **a** Schematic representation of the Ca_V_2.1 α_1_ subunit with the approximate location of the Proline 2455 Histidine (P2455H) mutation in the C-terminus. **b** Representative calcium current traces recorded from Ca_V_2.1α_1_ + 47/β_4_/α_2_δ-1 and P2455H + 47/β_4_/α_2_δ-1 channels. **c** Average current density–voltage relationship for cells expressing Ca_V_2.1α_1_ + 47/β_4_/α_2_δ-1 or P2455H + 47/β_4_/α_2_δ-1 channels. **d** Normalized activation curves. Inset: mean voltage for half activation for Ca_V_2.1α_1_ + 47/β_4_/α_2_δ-1 or P2455H + 47/β_4_/α_2_δ-1 channels (**p = 0.0077, two-tailed Student’s t test). **e** Average peak current density for whole cell calcium currents recorded from cells expressing Ca_V_2.1α_1_ + 47/β_4_/α_2_δ-1 or P2455H + 47/β_4_/α_2_δ-1 channels (p = 0.8914, two-tailed Student’s t test). **f** Time constant of current activation (τ_act_) as a function of voltage (*p < 0.05; **p < 0.01 vs Ca_V_2.1α_1_ + 47/β_4_/α_2_δ-1, two-tailed Student’s t test). Data are represented as mean ± SEM. Numbers in parentheses represent the number of cell recordings per variant

A recent study reported that patients with familial TN exhibit rare variants in ion channels, including the Ca_V_2.1 voltage gated calcium channel [4]. Ca_V_2.1 encodes native P-and Q-type calcium channels [5]. These channels are expressed at high levels in trigeminal neurons, the trigeminal sensory nuclear complex, the brain stem, cerebral cortex and cerebellum, where their main function is to control the release of neurotransmitters [6, 7]. The pore forming Ca_V_2.1 α_1_ subunit encompasses four major transmembrane domains, each containing six transmembrane segments termed S1-S6. The S4 segment senses membrane potential changes, whereas the linker between S5 and S6 lines the pore of the channel. Cytoplasmic regions connecting the various domains are involved in channel regulation, as are the N- and C-termini (Fig. 1a). The latter is a key site for calcium regulation of Ca_V_2.1 channel activity [8]. The Ca_V_2.1 α1 subunit associates with ancillary α_2_δ and β subunits to form a functional channel [7]. Multiple splice isoforms of Ca_V_2.1 have been reported in the literature [9].

Mutations in the *CACNA1A* gene (which encodes Ca_V_2.1) have been identified in patients with other forms of craniofacial pain, including familial hemiplegic migraine type 1 (FHM-1) [1, 6, 10]. A new missense mutation (P2455H) identified by Di Stefano and colleagues appears to be the first Ca_V_2.1 calcium channel mutation to be associated with a patient with classical TN [4]. Here we use functional expression in heterologous systems along with electrophysiological measurements to demonstrate that this variant causes a gain-of-function of channel activity.

## Materials and methods

### Molecular cloning

The wild-type human Ca_V_2.1 α1 subunit (+ 47 exon; NM_023035.1) clone in pcDNA3.1 Zeo (+) was kindly provided by Dr. Terrance Snutch (University of British Columbia, Vancouver, BC, Canada). Site-directed mutagenesis was performed using PCR (Pfu polymerase) along with forward and reverse mutagenesis primers for insertion of the P2455H mutation. After mutagenesis, the construct was analyzed by DNA sequencing.

### Cell culture and transfection

Human embryonic kidney tsA-201 cells were cultured and transiently transfected using the calcium phosphate method. Cells were transfected with 3 µg of hCa_V_2.1α_1_ + 47 or P2455H + 47 in combination with the auxiliary subunits β_4_ and α_2_δ-1, and 0.5 µg of eGFP to identify transfected cells.

### Electrophysiology recordings

Whole-cell voltage-clamp recordings were performed at room temperature (22–24 °C) 72 h post transfection. Currents were recorded using an Axopatch 200B amplifier linked to a computer with pCLAMP9.2 software. The external solution contained (in mM): 2 CaCl_2_ or 5 BaCl_2_, 137 or 132.5 CsCl, 1 MgCl_2_, 10 HEPES and 10 glucose (pH 7.4 adjusted with CsOH). Patch pipettes were filled with an internal solution containing (in mM): 130 CsCl, 2.5 MgCl_2_, 5 EGTA, 10 HEPES, 3 Na-ATP and 0.5 Mg-GTP (pH 7.4 adjusted with CsOH).

Peak current was determined by applying 250 ms pulses between − 60 mV and + 30 mV in 5 mV increments from a holding potential of − 100 mV to obtain the current–voltage (*I*–V) relation. The current density–voltage relationship was obtained by dividing the peak current at each voltage by the cell capacitance. The *I*–*V* curves were fitted using the following Boltzmann equation: (I = ((*V*_mem_–*V*_rev_) × *G*_max_)/(1 + exp((*V*_mem_–*V*_h*, *act_)/d*x*)). Where *I* is the peak current, *V*_mem_ is the membrane potential, *V*_rev_ is the reversal potential, *G*_max_ is the maximum conductance, *V*_h, act_ is the half voltage for activation, and d*x* is the slope factor. Time constants of activation (*τ*_act_) were obtained by mono-exponential fits to the late rising phase of the current using the following equation: *I* = *A *× (1 − exp(− *t*/*τ*)), where *A* is the amplitude of the current, and *τ* is the time constant. Activation curves were obtained from the *I*–*V* curves by dividing the peak current at each depolarizing step by the driving force according to the equation: *G* = *I/*(*V*_mem_ − *V*_rev_) and normalized against the maximum conductance (*G*_max_).

Steady-state inactivation curves were obtained by applying 5 s conditioning pre-pulses from − 80 mV to + 30 mV in 5 mV increments followed by a 140 ms test pulse to − 5 mV. Curves were fitted with the equation: *I*/*I*_max_ = 1/(1 + exp(− (*V*_mem_− V_h, inact_)/d*x*)) where *V*_h, inact_ is the half inactivation potential*.* Inactivation kinetics were determined by measuring the current remaining at the end of a 240 ms depolarization. Recovery from inactivation was evaluated by applying two test pulses, P1 (2 s) and P2 (5 ms), at 0 mV that were separated by an interval ranging from 20 ms to 7.5 s with a holding potential of − 100 mV. P2/P1 was plotted as a function of time.

### Data analysis

Data were analyzed using Clampfit 10.3 software (Molecular Devices) and fitted using GraphPad Prism 8. All averaged data are plotted as mean ± SEM. Statistical analysis was performed using a two-tailed Student’s t test and p < 0.05 was considered significant.

## Results and discussion

We characterized the influence of the P2455H mutation (Fig. 1a) on Ca_V_2.1 channel activity in a bath solution containing Ca^2+^ as the carrier ion. Representative traces for each variant are shown in Fig. 1b and reveal an apparent slowing of inactivation kinetics in the mutant channel. Figure 1c shows the current density–voltage relations for wild type and mutant Ca_V_2.1, revealing that the mutant exhibited a depolarizing shift in the voltage-dependence of activation (Ca_V_2.1: − 15.02 ± 1.36 mV; P2455H: − 10.45 ± 0.88, p = 0.0077, see also Fig. 1d). Furthermore, no difference was observed on peak current between both groups (Fig. 1e), and no change in cell surface expression of P2455H was observed when assayed via cell surface biotinylation (data not shown). The P2455H mutation mediated a slowing of the time course for activation (Fig. 1f).

**Fig. 2:**
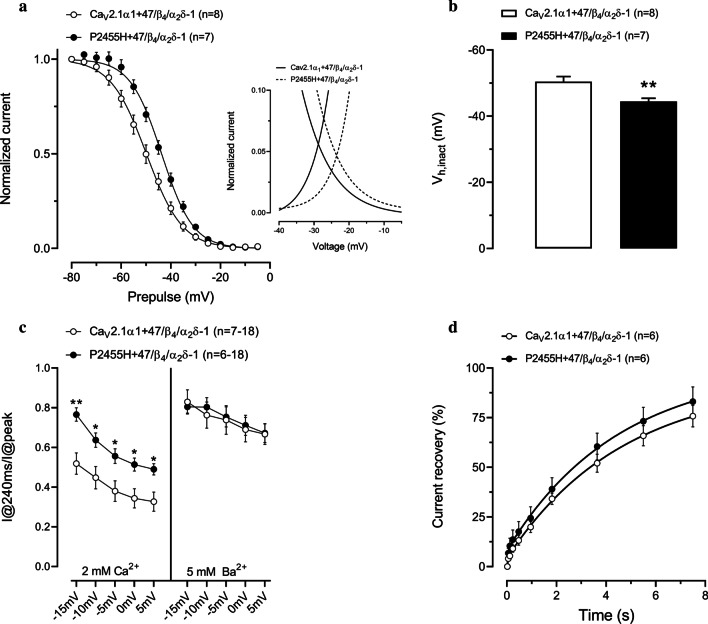
Functional effect of the P2455H mutation on Cav2.1 inactivation. **a** Steady-state inactivation curves for Ca_V_2.1α_1_ + 47/β_4_/α_2_δ-1 or P2455H + 47/β_4_/α_2_δ-1 channels. Inset: overlap of the activation and inactivation curves to illustrate the size and position of the window current. **b** Mean voltage for half inactivation for Ca_V_2.1α_1_ + 47/β_4_/α_2_δ-1 or P2455H + 47/β_4_/α_2_δ-1 channels (**p = 0.0073, vs Ca_V_2.1α_1_ + 47/β_4_/α_2_δ-1, two-tailed Student’s t test). **c** Fraction of current remaining at the end of a 240 ms depolarizing pulse using Ca^2+^ or Ba^2+^ as a charge carrier (*p < 0.05 and **p = 0.0076, two-tailed Student’s t test). **d** Comparison of the time course of recovery from inactivation for Ca_V_2.1α_1_ + 47/β_4_/α_2_δ-1 and P2455H + 47/β_4_/α_2_δ-1 channels. Data are represented as mean ± SEM. Numbers in parentheses represent the number of cells recordings per variant

Inspection of steady-state inactivation curves reveals that the mutant channel exhibits a depolarizing shift in the half inactivation potential (Fig. 2a, b), indicating a greater availability for opening (Ca_V_2.1: − 50.44 ± 1.53 mV; P2455H: − 44.45 ± 0.98 mV, p = 0.0073). As a consequence of the effects of the mutation on the voltage-dependences of activation and inactivation, the peak of the window current is shifted to more depolarizing potentials (Fig. 2a inset). We also assessed inactivation kinetics of the channel at multiple test potentials by measuring the fraction of current remaining at the end of a 240 ms depolarization. Figure 2c (left) shows that the P2455H exhibits slowed inactivation kinetics across the studied voltage range. To distinguish whether this effect was due to alterations in Ca^2+^- or voltage-dependent inactivation, we performed a set of recordings with Ba^2+^ as a charge carrier (Fig. 2c, right). When Ca^2+^ was removed, the difference in the inactivation kinetics between the wild type and mutant channel was abolished, indicating that the mutation affects Ca^2+^-dependent inactivation, rather than voltage-dependent inactivation kinetics. There was no difference in the time course of recovery from inactivation (Fig. 2d).

Collectively, our findings indicate that the P2455H mutation identified in a patient with TN [4] mediates compromised voltage-dependent activation, compromised Ca^2+^-dependent inactivation, and compromised steady-state inactivation of Ca_V_2.1. It is interesting to note that pathogenicity algorithms do not predict a pathogenic role of this variant, and yet, there are clear changes in the gating properties. This shows the importance of conducting functional analyses. Mutations in the C-terminal region of Ca_V_2.1 channel have already been described in other pathologies including FHM-1, episodic ataxia type II and spinocerebellar ataxia type 6 [10]. Of particular note, many of the characterized FHM-1 mutations lead to a gain-of-function in channel activity [6], in some cases in a channel splice isoform dependent manner [11]. Here we report that the TN mutation P2455H also causes a gain-of-function by inhibiting inactivation, but at the same time a loss of function due to reduced activation gating. How these alterations in biophysical changes manifest themselves physiologically may depend on different subtypes of neurons that are present in trigeminal ganglia [12]. For example, gain-of-channel function due to slowing of inactivation may have a more profound impact in neurons with high frequency firing, whereas the depolarizing shift in activation and the associated reduction of function may be more important in neurons with lower firing rates. Along these lines, it is known that gain-of-function mutations in Ca_v_2.1 channels have been associated with conditions such as FMH-1 [13] due to a hyperpolarizing shift in the half activation voltage of the channel that enhances excitatory synaptic transmission and leads to cortical spreading depression. The gain-of-function observed here affects predominantly the inactivation properties of the channel, thus perhaps accounting for the absence of FHM-1 in this patient. We also note that the pathology in the affected patient became evident after age 46 [4]. In this context, we note that the patient’s MRI also showed a neurovascular compression [4], and it is thus possible that the effect of the mutation only manifested itself after this additional physical injury.

It is interesting to note that amino acid substitution drastically reduced Ca^2+^-dependent inactivation, even though the mutated residue lies downstream of the pre-IQ and IQ motifs that are known to interact with calmodulin and are critical for this type of inactivation (Fig. 1a) [14, 15]. These data thus reveal a previously unrecognized role of the distal Ca_V_2.1 C-terminus region in calcium regulation of Ca_V_2.1. The molecular basis of this effect remains to be determined, although it is worth noting that the distal C-terminus in Ca_V_1.4 channels has been shown to mediate an auto-inhibition of Ca^2+^-dependent inactivation [14, 16]. Ca_V_2.1 plays a critical role in neurotransmitter release, and hence, a gain-of-function of channel activity is consistent with enhance synaptic transmission in the trigeminal system [14, 15]. Furthermore, the C-terminal region of Ca_v_2 channels is known to be involved in the tethering of synaptic vesicles [17] and it is thus possible that the mutation may interfere with this process. Collectively, this may explain the increase in the affected individual’s nociceptive processing.

In summary, to our knowledge, the P2455H variant was the first Ca_V_2.1 channel mutation to be associated with trigeminal neuralgia. Our data demonstrate that this new missense variant in the *CACNA1A* gene mediates both gain and loss of function effects in Cav2.1 that are expected to affect signal processing in the trigeminal system.

## Data Availability

All data generated or analyzed during this study are included in this published article.

## Abbreviations

TN: Trigeminal neuralgia
FMH-1: Familial hemiplegic migraine type 1
